# Initial experience with the providence nighttime bracing in adolescent idiopathic scoliosis

**DOI:** 10.1186/1748-7161-9-S1-O33

**Published:** 2014-12-04

**Authors:** Inge Beuschau, Lena Quisth, Ane Simony

**Affiliations:** 1 ORTOS, Odense, Denmark; 2 Sector for Spine Surgery and Research, Middelfart Hospital, Denmark

## Background

Nearly six years ago the primary conservative treatment of adolescent idiopathic scoliosis (AIS) in the southern part of Denmark, went from full time bracing and hospitalization to nighttime bracing and an in- and outpatient treatment.

## Aim

To evaluate the effectiveness of nighttime bracing in AIS.

Methods: Inclusion criteria were patients diagnosed with AIS and skeletal immature. With an apex of the primary curve from TH7 and below and with a Cobb angel between 20- 45 degrees. The patients were asked to wear the brace at least 7-8 hours pr. night.

No other previous treatments were accepted and a follow up at least 6 months out of brace. The brace treatment was continued until two years post menarche or for male at the expected adult height.

Cross-measured x-rays were used to compare the primary Cobb angel, the in-brace correction and the outcome Cobb angel. A decreased outcome Cobb angle as well as the overcorrection of the curve measured in brace was recorded as zero.

The brace treatment was considered failed if progression > 5 degrees occurred and if surgery were performed.

## Results

A total of 55 patients, 8 male and 47 female, with a mean age at 14 years (11-16.5) and the mean primary Cobb at 31 degrees (20-41) were included in this study. There were 27 primary thoracic curves, 16 thoracolumbar, 11 lumbar and 1 double curve.

The mean time of treatment was 18 month (5-59).

The average in-brace correction was 81% (24-100%), with a mean in-brace Cobb of 6,1 degrees (0-26). After ended treatment the mean Cobb angle was 28 degrees (7-50), an average of no progression. The end results were 11 failures (6-15 degrees); equal 20 % and out of these 11 patients, 3 had surgery performed (5%).

## Conclusion

The results show a good curve control and an acceptable 20 % failure rate, which is equal to other studies. The providence brace is an excellent alternative to standard conservative treatment. Larger studies are needed to establish the relationship between in-brace correction and curve progression during the treatment.
